# Professor Robert Koch

**Published:** 1910-07

**Authors:** 


Professor Robert Koch, the bacteriologist, died at Baden-Baden, Germany, May 27, 1910, from a disease of the heart. He was born at Clausthal, Hanover, December 11, 1843. Professor Koch became distinguished as an investigator of microörganisms, but probably gained most renown as the discoverer of the bacilli of tuberculosis and cholera. It was in 1882 that he announced his discovery of the bacillus of tuberculosis. The following year he was sent by the German Government to India and Egypt to study cholera and discovered the comma bacillus, the presence of which is regarded as an infallible test in diagnosticating Asiatic cholera.

